# Dioscin *elevates* lncRNA MANTIS in therapeutic angiogenesis for heart diseases

**DOI:** 10.1111/acel.13392

**Published:** 2021-06-03

**Authors:** Chuiyu Kong, Dayin Lyu, Chang He, Rui Li, Qiulun Lu

**Affiliations:** ^1^ Key Laboratory of Cardiovascular and Cerebrovascular Medicine School of Pharmacy Nanjing Medical University Nanjing China

**Keywords:** angiogenesis, Dioscin, MANTIS, myocardial infarction

## Abstract

Dioscin has been widely used in clinics for coronary artery disease (CAD) treatment for years in China. However, the underlying mechanism for Dioscin‐mediated cardioprotective effect has not been elucidated. Here, we showed that Dioscin significantly rescues the cardiac function in mouse model of myocardial infarction (MI), accompanied by the reduction of cardiac fibrosis and apoptosis, resulting from elevated angiogenesis. Mechanistically, Dioscin promotes the proliferation and migration of hypoxic endothelial cells *via* the up‐regulation of lncRNA *MANTIS*, which serves as a scaffolding lncRNA within a chromatin remodeling complex. Meanwhile, it enables pol II binding to the transcription start sites, which leads to induced expression of angiogenesis‐related genes, including SOX18, SMAD6, and COUP‐TFII. Conversely, IncRNA *MANTIS* silencing prevents Dioscin‐induced migration and angiogenesis in hypoxic endothelial cells. Taken together, these data provide new insights that clarifies the cardioprotective effects of Dioscin against myocardial infarcted injury and confirms the effect on angiogenic activity of endothelial cells. This will build a solid theoretical basis for clinical therapeutic strategies.

## Graphical abstract

Dioscin alleviates cardiac dysfunction in infarcted hearts *via* lncRNA MANTIS regulation. Dioscin promotes MANTIS and BRG1 interaction, resulting in angiogenic promotion and cardiac fucntion recovery.

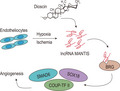

## 1 INTRODUCTION

The major function of the circulatory system is to deliver oxygen and nutrients to the cells throughout body (Del Re et al., 2019; Kaelin et al., 2016). An insufficient blood supply, if not corrected in time, leads to depletion of oxygen and nutrients to vital organs, resulting in organ failure and death. As we know, coronary artery disease (CAD) and stroke caused by ischemia, a sudden and severe blockage of coronary or brain arteries, are one of the most common causes of death worldwide (Khera & Kathiresan, 2017; Vogel et al., 2019). Every year, an estimation of 720,000 people in the United States is hospitalized with acute coronary syndrome or has fatal coronary heart disease events (Rodriguez & Harrington, 2021).

Myocardial infarction (MI) is the most severe manifestation of CAD. Approximately every 40 s, an individual is hospitalized due to myocardial infarction (van Diepen et al., 2017). Reperfusion strategies, including angiography and percutaneous coronary intervention (PCI), are the widely used for CAD and MI patients (Thiele et al., 2018). Also, artery bypass graft surgery is considered to treat MI patients with a large myocardial area at jeopardy or cardiogenic shock (Solo et al., 2019). However, these therapeutic strategies are not suitable for some elder patients with diffuse myocardial infarction. To meet the needs for those patients, therapeutic angiogenesis has been proposed as an attractive novel strategy for CAD and MI treatment. It is a combined utilization of chemicals and angiogenic factors to enhance neovascularization and growth of collateral blood vessels, which act as endogenous bypass conduits to increase tissue perfusion and provide oxygen and nutrients in the ischemic area. Thus, it is an emerged demand to identify novel and effective angiogenic compound for MI treatment.

Dioscin, a glucoside saponin, is one natural product identified from certain medicinal plants such as *Dioscoreazingiberensis* Wright and *Dioscoreanipponica* Makino (Tao et al., 2018). Recent studies suggest that Dioscin lowers the lipid levels and represses the inflammatory response, showing the hepatoprotective effects (Yao et al., 2018; Zhang et al., 2017). Additionally, it has been widely used in clinics as CAD treatment for many years in China. However, the mechanism underlying the protective effect of Dioscin on the infarcted hearts needs to be elucidated.

Recently, emerging data indicate that long noncoding RNAs (LncRNA, >200 nucleotides in length), a major population of transcriptome, play critical roles in the physiology and pathophysiology of cardiovascular diseases by distinct mechanisms (Ransohoff et al., 2018). Hypoxia‐induced endothelial lncRNA *MANTIS* (metastasis‐associated lung adenocarcinoma transcript 1) identified in the antisense strand of the intron of Annexin A4, a calcium and phospholipid binding protein, is expressed in different cellular types. *MANTIS* silencing causes the endothelial dysfunction, impairing sprouting and tube formation, and attenuating endothelial migration. *MANTIS* is required for endothelial cells proliferation, neonatal retina vascularization, and vascular growth in vivo after hindlimb ischemia (Cremer et al., 2019). A key function of lncRNA *MANTIS* is by interacting and combining with brahma related gene‐1 (BRG1), to regulate gene transcription, including SRY (sex determining region Y)‐box 18 (SOX18), mothers against decapentaplegic homologue 6 (SMAD6), and nuclear receptor subfamily 2 group F member 2 (COUP‐TFII), which are important for angiogenesis in endothelial cells. Although lncRNAs have been proven crucial in ischemic diseases, whether lncRNAs are regulated by Dioscin remains unclear in myocardial infarction.

Here, we demonstrate that a natural product Dioscin alleviates hypoxic‐caused cardiac dysfunction in mouse model of myocardial infarction *via* up‐regulating the level of lncRNA *MANTIS*. We show that Dioscin increases IncRNA *MANTIS* expression, promoting the complex formation of *MANTIS* and BRG1, elevating the expression of SOX18, SMAD6, and COUP‐TFII, resulting in promoted angiogenesis in myocardial infarction.

## 2 RESULTS

### 2.1 Dioscin attenuates cardiac dysfunction in mouse model of myocardial infarction

**FIGURE 1:**
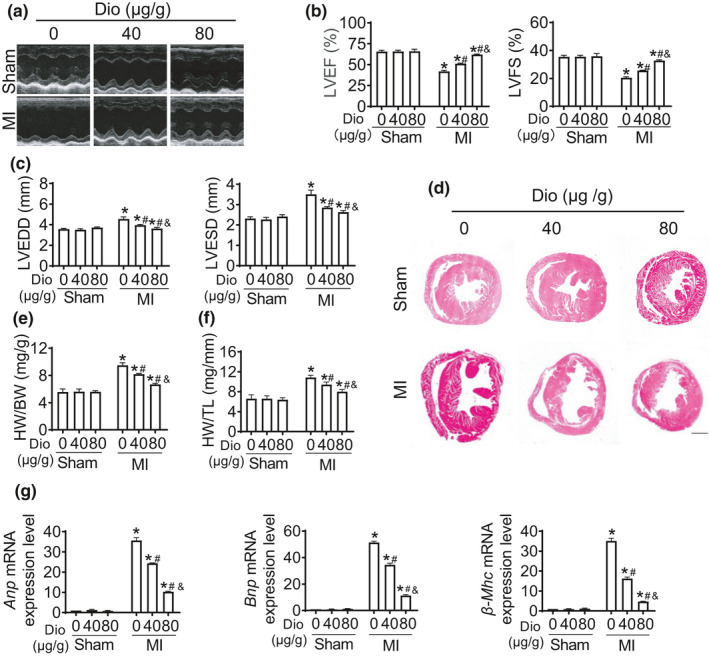
Dioscin protects cardiac function in mouse model of myocardial infarction. 8 weeks old mice were intragastric administrated with Vehicle (Veh) or Dioscin (Dio) for 2 weeks after myocardial infarction (*n* = 6 each group). (a) Representative echocardiogram of the cardiac function of mice (M‐mode, parasternal short axis) for each condition. (b) Quantification of left ventricular ejection fraction (LVEF, %) and left ventricular fractional shortening (LVFS, %). (c) Quantification of left ventricular end‐diastolic internal dimension (LVEDD, mm) and left ventricular end‐systolic internal dimension (LVESD, mm). (d) Hematoxylin and eosin (H&E) staining (scale bar: 1mm) of heart sections from mice treated with Vehicle or Dioscin. (e,f) Ratios of heart weight to body weight (HW/BW) and heart weight to tibia length (HW/TL) were shown, respectively. (g) Real‐time PCR analysis of the messenger RNA (mRNA) expression of *Anp*, *Bnp*, and *β*‐*Mhc* was performed using heart tissues from Sham group and MI group treated with Vehicle or Dioscin. Data were presented as mean ± SEM. *p < 0.05 relative to Sham; #*p* < 0.05 relative to MI; &*p* < 0.05 relative to MI with 40 μg/g Dioscin

To explore the effects of Dioscin in response to MI, we applied Dioscin as a therapeutic treatment in mouse MI model. Echocardiography showed that Dioscin significantly improved cardiac functions after MI (Figure 1a). Ejection fraction (EF), a vital parameter for cardiac function, was improved after Dioscin treatment in infarcted hearts, compared to vehicle treatment (Figure 1b). Similarly, significant improvement was observed for fraction shortening (FS) with Dioscin administration (Figure 1b). Additionally, declination of the left ventricular end‐diastolic diameters (LVEDD) and left ventricular end‐systolic diameters (LVESD) in response to MI was dramatically rescued by Dioscin in mice suffered with MI, indicating Dioscin could help to maintain cardiac structure in infarcted hearts (Figure 1c).

Dioscin repressed the increase of heart size caused by myocardial infarction (Figure 1d). As shown in Figure 1e,f, MI caused the increase of the ratios of heart weight (HW) to body weight (BW) or tibia length (TL). However, this hypertrophic tendency was alleviated by Dioscin administration. Additionally, the mRNA levels of biomarkers for heart failure, including *Anp*, *Bnp*, and *β*‐*Mhc*, were declined after MI with Dioscin treatment (Figure 1g). These findings suggest that Dioscin can rescue cardiac function and delay the progress of heart failure caused by myocardial infarction.

### 2.2 Dioscin promotes angiogenesis in mice after myocardial infarction

**FIGURE 2:**
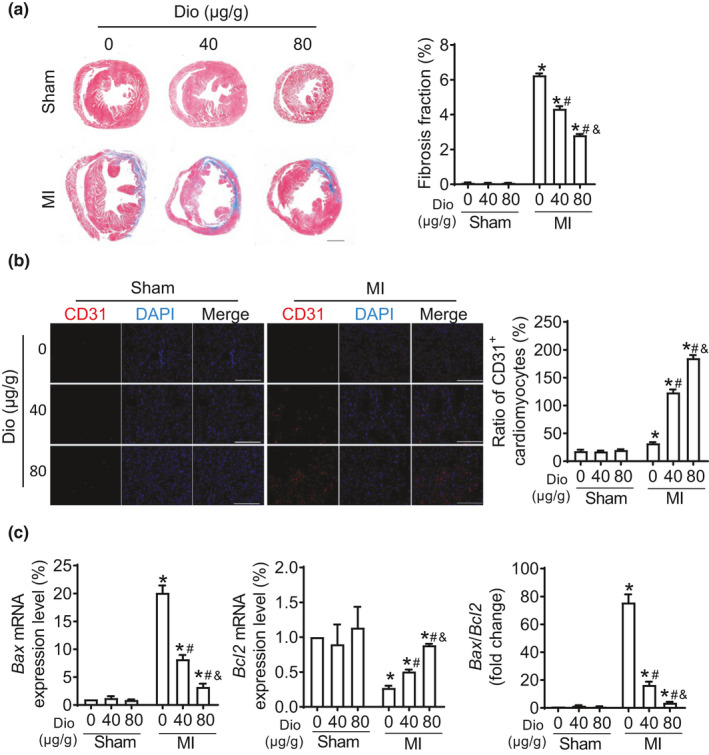
Dioscin promotes angiogenesis in MI mouse model. (a) Representative Masson trichrome staining from heart tissues (scale bar: 1mm) and their quantification. (b) Representative immunofluorescent images were staining with CD31 (red) using heart tissues from mice (scale bar: 100 μm), and the quantification was showed at the right. (c) Real‐time PCR assays for the ratio of *Bax*/*Bcl_2_* mRNA levels were performed. Date were expressed as mean ± SEM. *p < 0.05 relative to Sham; #*p* < 0.05 relative to MI; &*p* < 0.05 relative to MI with 40 μg/g Dioscin

To further confirm whether Dioscin attenuates the cardiac dysfunction after MI, we explored the therapeutical effect of Dioscin in the infarcted hearts. Administration of Dioscin significantly reduced the size of cardiac fibrosis in response to MI, compared to vehicle (Figure 2a). The number of CD31‐positive cells was increased in Dioscin‐treated infarcted hearts compared with vehicle treatment, indicating that Dioscin promoted angiogenesis in mouse model of MI (Figure 2b). What is more, the ratio for *Bcl2*/*Bax* mRNAs was declined in infarcted hearts, and this reduction was blocked by Dioscin (Figure 2c). These data indicate that Doscin enhances angiogenesis and alleviates cardiac apoptosis and fibrosis in response to infarction.

### 2.3 Dioscin enhances angiogenesis in hypoxic endothelial cells

**FIGURE 3:**
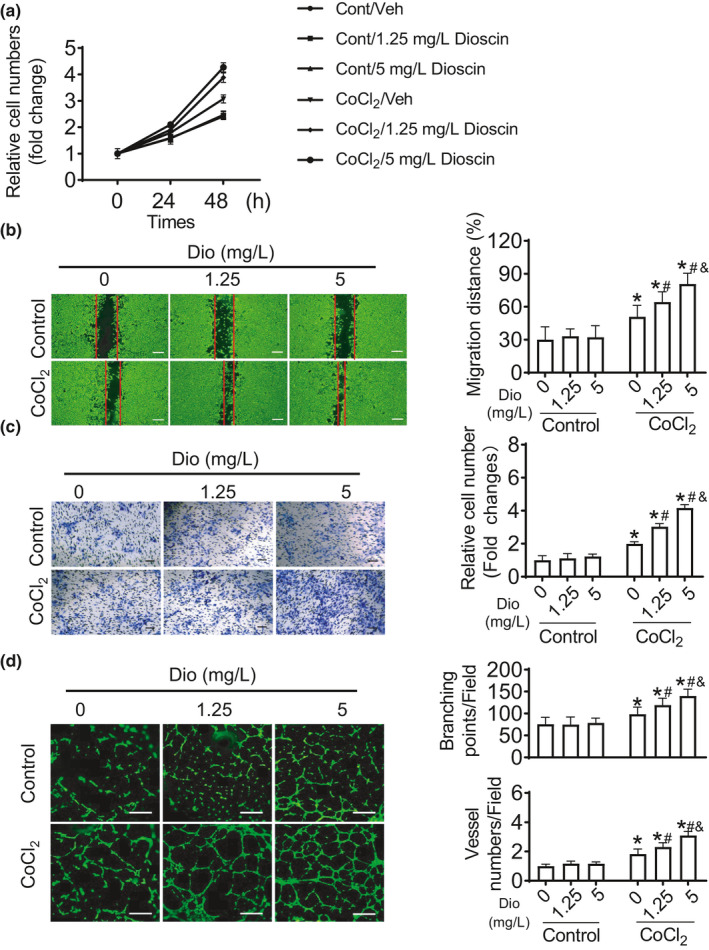
Dioscin promotes angiogenesis in hypoxic endothelial cells. (a) Dioscin promoted ECs proliferation in hypoxic condition. (b) Scratch assays were performed to measure the migration ability for Dioscin (scale bar: 500 μm). (c) Dioscin induced endothelial migration using transwell migration assays (scale bar: 200 μm). (d) Dioscin enhanced tube formation (scale bar: 100 μm). Date were expressed as mean ± SEM. *p < 0.05 relative to Control; #*p* < 0.05 relative to CoCl_2_ with Vehicle; &*p* < 0.05 relative to CoCl_2_ with 1.25 mg/L Dioscin

Although angiogenesis is associated with Dioscin treatment in mouse model of MI, whether Dioscin promotes angiogenesis or not is uncovered. *In vitro* experiments, human umbilical vein endothelial cells (HUVECs) were incubated with cobalt (II) chloride (CoCl_2_) to mimic hypoxic condition. Under basic condition, Dioscin did not affect the endothelial proliferation (Figure 3a). Notably, in hypoxic condition, the number of CoCl_2_‐cultured endothelial cells was increased, accompanying with the increased doses of Dioscin (Figure 3a). In wound healing assays, Dioscin promoted endothelial cell migration in dose‐dependent manner under hypoxic condition (Figure 3b). Consistently with the results from wound healing assays, in response to hypoxia, Dioscin induced more ECs migrated across the membrane in transwell migration assays (Figure 3c). We also performed tube formation assays, showing that Dioscin increased the numbers for capillary tubes and branching points in hypoxic condition (Figure 3d). These data suggest that Dioscin promotes angiogenesis in hypoxic endothelial cells.

### 2.4 Increase for lncRNA *MANTIS* is associated with Dioscin treatment

**FIGURE 4:**
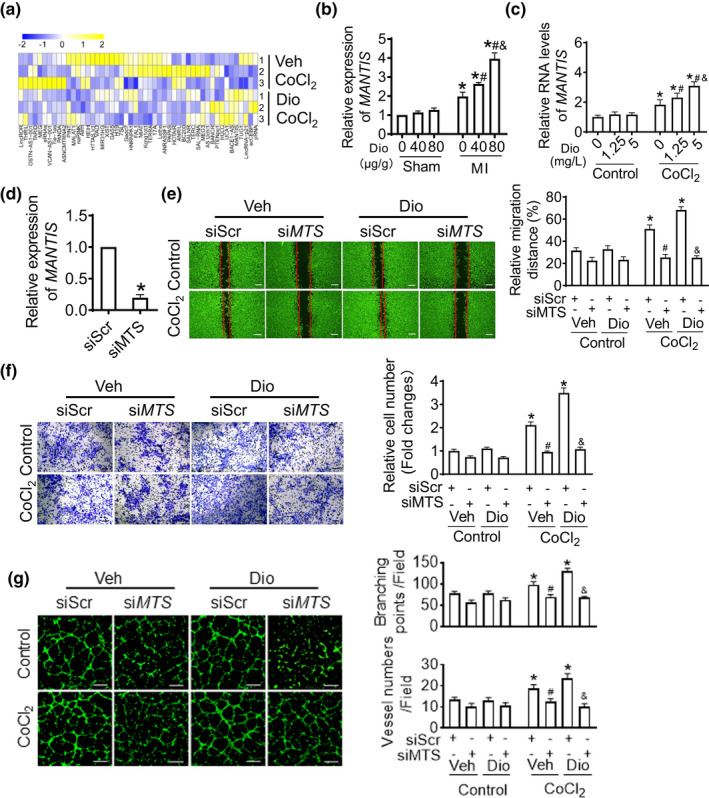
Dioscin increases IncRNA *MANTIS* level in hypoxic endothelial cells. (a) The expression levels for lncRNAs were detected in Dioscin‐treated hypoxic endothelial cells by using real‐time PCR assays. (b) Dioscin promoted expression of *MANTIS* in infarcted heart tissue. (c) Dioscin promoted expression of *MANTIS* in endothelial cells. (d) The efficiency of si*MANTIS* in endothelial cells. (e) *MANTIS* knockdown inhibited Dioscin‐induced migration of ECs using scratch assays (scale bar: 500 μm). (f) Transfection of siRNA against *MANTIS* inhibited Dioscin‐induced migration of ECs using transwell assays (scale bar: 200 μm). (g) *MANTIS*‐specific siRNA inhibited Dioscin‐induced tube formation of ECs (scale bar: 100 μm). Date were expressed as mean ± SEM. *p < 0.05 relative to Control; #*p* < 0.05 relative to CoCl_2_ with Vehicle; &*p* < 0.05 relative to CoCl_2_ with 1.25 mg/L Dioscin [Corrections added on 30 June 2021 after first online publication: the 4th image in the first upper‐lane in Figure 4g (DIO treatment with siMTS) was incorrect and also CoCl_2_ in the lower graph was incorrectly displayed as CoCl. These errors were corrected and revised figure was updated in this version.]

As lncRNAs are involved in the development of cardiovascular diseases, we examined whether angiogenesis is involved in regulating the expression of lncRNAs that were reported in endothelial cells. We profiled 50 lncRNAs, reported which play a vital role in endothelial cells, in hypoxic HUVECs with/without Dioscin treatment using real‐time PCR assays (Figure 4a). As depicted in Figure 4a, the change for lncRNA *MANTIS* expression was highest after Dioscin treatment. To confirm these results, the levels for *MANTIS* were detected in different doses of Dioscin, showing the expression level of *MANTIS* was positively correlated with the doses of Dioscin both in infarcted heart tissue and hypoxic HUVECs (Figure 4b,c).

### 2.5 Dioscin induces angiogenesis *via* up‐regulation of lncRNA *MANTIS* in hypoxic endothelial cells

In order to identify whether Dioscin promotes angiogenesis *via* *MANTIS* or not, we detected angiogenesis after *MANTIS* knockdown in Dioscin‐treated HUVECs. First, we detected the efficiency of si*MANTIS*, which decreased about 70% in mRNA level (Figure 4d). In the scratch assays, although Dioscin promoted the migration of hypoxic endothelial cells into the wounded areas, deficiency of *MANTIS* abolished endothelial migration caused by Dioscin (Figure 4e). Additionally, in transwell migration assays, Dioscin increased the numbers of ECs that migrated across the membranes, and this increase was suppressed after *MANTIS* knockdown (Figure 4f). Consistently, the capillary tube formation mediated by Dioscin was alleviated by the reduction of *MANTIS* (Figure 4g). Taken together, these results suggest that Dioscin mediates the effect of angiogenesis *via* up‐regulation of *MANTIS*.

### 2.6 Dioscin accelerates the formation of *MANTIS* and BRG1 complex

**FIGURE 5:**
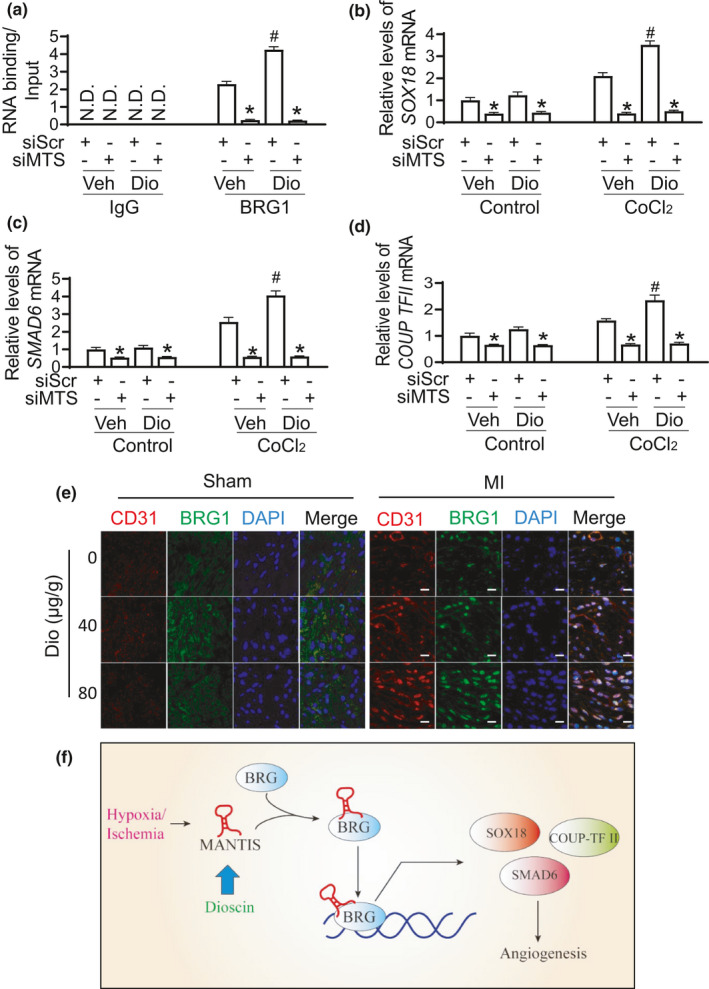
Dioscin enhances the complex formation between *MANTIS* and BRG1. (a) Chromatin Immunoprecipitation (CHIP) was performed to access the assembly of the *MANTIS* and BRG1 by Dioscin. (b) Real‐time reverse transcription (RT‐PCR) analysis was performed for *SOX18* mRNA expression. (c) *SMAD6* mRNA level was detected in Dioscin‐treated endothelial cells using real‐time PCR analysis. (d) Real‐time PCR analysis for *COUP*‐*TFII* mRNA expression. (e) Representative images of CD31 (red), BRG1 (green), and nucleus (blue) co‐staining in endothelial cells in heart tissue after Sham and MI group (scar bar: 50 um). (f) Schematic diagram showing the molecular signing pathway for Dioscin‐induced angiogenesis. *p < 0.05 relative to siScr; #*p* < 0.05 relative to Vehicle

It was reported that *MANTIS* can interact with BRG1, regulating angiogenic gene expression. To assess whether Dioscin regulates an interaction of BRG1/*MANTIS* complex for angiogenesis or not, we performed RNA chromatin immunoprecipitation assays (RNA ChIP) in hypoxic condition. In HUVECs, hypoxia promoted the formation of BRG1 and *MANTIS*, while Dioscin accelerated this complex formation (Figure 5a).

To further verify Dioscin affects angiogenesis *via* the complex of BRG1 and *MANTIS*, we measured the expression levels for BRG1 downstream, such as SOX18, SMAD6, and COUP‐TFII. The increase for these genes expression was observed after Dioscin treatment. However, *MANTIS* knockdown repressed the increase for SOX18, SMAD6, and COUP‐TFII expression (Figure 5b–d). Furthermore, the co‐staining for CD31 and BRG1 strongly demonstrated that the expression of BRG1 was significant enhanced in endothelial cells after Dioscin treatment in MI model (Figure 5e). These data indicate that Dioscin enhances angiogenesis *via* the formation of *MANTIS* and BRG1 complex (Figure 5f).

## 3 DISCUSSION

In this study, we demonstrated that Dioscin efficiently rescues cardiac dysfunction in response to myocardial infarction. Interestingly, cardioprotective Dioscin promotes the increase of lncRNA *MANTIS*, formatting the complex of *MANTIS* and BRG1, resulting in tube formation and angiogenesis, alleviating cardiac fibrosis and apoptosis. Our results suggest that Dioscin is a novel candidate for therapeutic strategy for MI.

Dioscin has a potential effect of angiogenesis and anti‐apoptosis on infarcted hearts. Currently, United States Food and Drug Administration (FDA) has not approved any therapeutic angiogenic strategies for CAD/MI treatment (Bergers & Hanahan, 2008; Fischer et al., 2008). However, new angiogenic factors and chemicals are urgent to be identified and applied in clinics. In our study, Dioscin is considered as a potential and effective therapeutic nature product for infarcted hearts, rescuing the cardiac function in mouse model of MI *via* promoting of angiogenesis. Additionally, the cardiac fibrosis and apoptosis were alleviated by Dioscin in infarcted hearts. Moreover, Tao *et al* demonstrated that Dioscin alleviates cerebral inflammation *via* HMGB‐1/TLR‐4 signaling pathway after middle cerebral artery occlusion in mouse, preventing cellular apoptosis (Tao et al., 2015). Herein, the apoptotic cells were suppressed by Dioscin in MI mouse model, reflecting from the reduction of *Bax* and the augment of *Bcl2*. It is possible that the effect of Dioscin‐caused anti‐apoptosis is resulted from the promoted angiogenesis in ischemic hearts. Once angiogenesis occurs, the supply of nutritions and oxygen is recovered in the ischemic tissues, leading to suppress apoptosis (Esposito et al., 2018; Lin et al., 2018). Thus, Dioscin is a potent and promising candidate of therapeutic strategy for ischemic diseases.

Another major novelty of this work is identified that Dioscin promotes angiogenesis *via* up‐regulation of lncRNA *MANTIS*. *MANTIS* silencing leads to the endothelial dysfunction, impairing sprouting and tube formation, and attenuating endothelial migration (Zampetaki & Mayr, 2017). Down‐regulation of angiogenesis‐related genes was observed after *MANTIS* knockdown. Mechanistically, in endothelial cells, *MANTIS* has been found to act as a scaffolding lncRNA within a chromatin remodeling complex and up‐regulate endothelial genes, including SOX18, SMAD6, and COUP‐TFII transcription by enabling pol II binding to the transcription start sites (Michalik et al., 2014; Yan et al., 2015). It is of high interest that Dioscin increases the levels of *MANTIS* both in MI mouse model and hypoxic endothelial cells, accelerating endothelial proliferation and migration in ischemic/hypoxic conditions, resulting in therapeutic angiogenesis.

In summary, we show that Dioscin has a good cardioprotective effect against myocardial infarction in mice *via* the up‐regulation of lncRNA *MANTIS*, accelerating endothelial migration and angiogenesis, attenuating apoptosis and fibrosis, and resulting in alleviating cardiac dysfunction. Accordingly, Dioscin may serve as a novel treatment not only for myocardial infarction, but also for many other ischemic diseases.

## 4 MATERIALS AND METHODS

### 4.1 Mouse model

Male C57BL/6 mice were unrestricted access to food and water and were housed in the controlled environment, with regulation of temperature (22 ± 1°C) and humidity (55%), a 12: 12‐h dark‐light cycle. All animal experiments were carried out in accordance with the principles provided by the National Institute of Health Guideline and were approved by the Animal Care and Use Committee of Nanjing Medical University (IACUC‐1912034).

Mouse model of MI was generated by ligation of the left anterior descending (LAD) as described previously with male mice at the age of 8–10 weeks (Lu et al., 2016). Briefly, the mice were anesthetized with isoflurane and then intubated with a fine polyethylene cannula connected to a small animal ventilator. A thoracotomy incision was performed in the second intercostal space, and the heart was exteriorized out of the chest. The LAD coronary artery was ligated permanently with a 7–0 nonabsorbable surgical suture, and the heart was then returned inside the chest. The chest wall was closed in layers, and skin incision was closed by sutures. Mice with sham‐operation were subjected to the same surgical treatment, but the LAD was not ligated.

Dioscin (Di'ao group, Chengdu, China) was intragastric administration 1 day after ligation and continued until the mice were sacrificed 2 weeks. Mice in the control group received an equal volume of the solvent orally.

### 4.2 Transfections

Primary human umbilical vein endothelial cells (HUVECs) were isolated from the human umbilical vein, as previously described (Cai et al., 2015). The cells were cultured in the EGM medium containing 5% (v/v) fetal bovine serum (FBS) and EGM‐2 Single Quots (Lonza, USA).

Human umbilical vein endothelial cells were transfected with siRNA using the electroporation P5 Primary Cell Nucleofector Kits (Lonza). The transfection efficiency of siRNA was determined by real‐time RT‐PCR analysis of a target gene. The sequences of si*MANTIS*: sense, 5′‐AAA UGA AGC AGC CUU GUU GUC UGG G‐3′; anti‐sense, 5′‐AAA UCU GAA GCG ACG UUC UGU UGG G‐3′ (Genpharm, China).

### 4.3 RNA ChIP assay

Preparation of cell extracts, crosslinking, and isolation of nuclei was performed with the RNA ChIP‐IT (Active Motif, USA) according to the manufacturer' s protocol. After sonification of the lysates, cell debris was removed by centrifugation and the supernatant was digested with DNase I. After digestion, the samples were incubated with protein G magnetic beads, BRG1 antibody (IgG as control), and Protease inhibitor over night at 4°C. Elution of the beads was done with elution buffer, and then binding RNA was purified with TRIzol and digested with DNaes I. Samples were reverse‐transcribed as previously reported (Ge et al., 2014).

### 4.4 Scratch wound assays

Human umbilical vein endothelial cells were cultured as a confluent monolayer and scratched with a pipette tip. The cells were incubated with EBM medium and EGM‐2 Single Quots. HUVECs were incubated with/without Dioscin. 12 h later, 2 μg/ml of Calcein AM (Invitrogen, USA) was added directly to the well and incubated for 15 min. The images were visualized and captured.

Migration was quantified as the ratio of the area covered with cells to the cell‐free area.

### 4.5 Transwell assays

Approximately 5 × 10^4^ HUVECs were plated into the upper chamber and were allowed to migrate toward the lower plate (Corning Costar, USA). After 6 h of incubation, HUVECs on the bottom of the Transwell membrane were fixed with 4% paraformaldehyde at 37°C for 20 min and stained with hematoxylin. The membranes were washed three times with PBS and photographed. The migrated cells on the bottom of the surface were counted in eight standardized fields.

### 4.6 Matrigel assays (tube formation)

Human umbilical vein endothelial cells in EBM were seeded onto Matrigel (Corning, USA) with Dioscin or negative control. After 8 h of incubation, 2 μg/ml of Calcein AM (Invitrogen, USA) was added directly to the well and incubated for 15 min. The images were visualized and captured. The number of vessel tubes formed was analyzed as described previously (Lu et al., 2016).

### 4.7 Real‐time RT‐PCR assays

Total RNA was isolated from mouse hearts or cultured cells using TRIzol (Invitrogen, USA) according to the manufacturer's instruction (Li et al., 2019). A total of 0.5 μg of RNA samples were reverse‐transcribed using M‐MLV Reverse Transcriptase, following the manufacturer's protocol (Promega, USA). Real‐time PCR assay was then performed using the FastStart Universal SYBR Green Master (Roche, Switzerland) and Real‐time PCR Detection System (Bio‐Rad, USA) as described previously. Experiments were performed in triplicate, and the relative expression levels of the genes were calculated using the 2^−ΔΔCT^ method. The primers used were listed in Table 1 and Table 2.

**TABLE 1:** Primers for quantitative reverse transcription PCR detection

Species	Name	Forward primer (5′−3′)	Reverse primer (5′−3′)
Human	*SOX18*	CATGGTGTGGGCAAAGGAC	GCCGGTACTTGTAGTTGGG
Human	*SMAD6*	CCTCTATGCGGTGTACGAC	GATGCCGAAGCCGATCTTG
Human	*COUP‐TFII*	CGCCTTTATGGACCACATACG	TCCACATGGGCTACATCAGAG
Human	*18S rRNA*	CTTTGGTCGCTCGCTCCTC	CTGACCGGGTTGGTTTTGAT
Human	*MANTIS*	AACTCCTGCTCCAAACTCACTC	CCAGAGACTTTCCATTCTGATG
Mouse	*Anp*	ACCTCCCGAAGCTACCTAAGT	CAACCTTTTCAACGGCTCCAA
Mouse	*Bnp*	GAGGTCACTCCTATCCTCTGG	GCCATTTCCTCCGACTTTTCTC
Mouse	*β‐Mhc*	GAGGGTGGCTCTCACACATTC	TTGGCCTTCGTAAGCAAACTG
Mouse	*Bax*	CCAAGAAGCTGAGCGAGTGT	CACGTCAGCAATCATCCTCTG
Mouse	*Bcl2*	TGGCATCTTCTCCTTCCAGC	ACGTCCTGGCAGCCATGTC
Mouse	*GAPDH*	ACAACTTTGGCATTGTGGAA	GATGCAGGGATGATGTTCTG

**TABLE 2:** Primers for quantitative reverse transcription PCR detection of IncRNA

Name	Forward primer (5′−3′)	Reverse primer (5′−3′)
LincROR	TATAATGAGATACCACCTTA	AGGAACTGTCATACCGTTTC
THRIL	AACTCCTGACCTCAGGTGATCCAT	AAGGGAGTTTCAGAAGGTGTGGCT
TARID	CGGTCACCACTTCTTTCAGG	CGTATGTGAAGACAAAGGCAAC
MEG	CTCCCCTTCTAGCGCTCACG	CTAGCCGCCGTCTATACTACCGGCT
VCAN‐AS1‐001	AGAGCATGTTTTCCTTGGCTTT	TATGTCAGCTGTGATGTGGCA
PANDA	TGCACACATTTAACCCGAAG	CCCCAAAGCTACATCTATGACA
ASNCMTRNA2	ACCGTGCAAAGGTAGCATAATCACT	CCGTAAATGATATCATCTCAACT
PINT	CGCGCACGTATTCTTGTATG	AGGAACCCGAAAGACACCTT
MALAT1	TTATCCTTGGAAGAGTATT	TAAGAAGTCACATTATTGG
HEIH	CCTCTTGTGCCCCTTTCT	AGGTCTCATGGCTTCTCG
HTTAS V1	GCGCCGCTCAGCAC	GCAAGGACAGTCTTTCTCTGATGTT
VAD	ACCTGAAAACTACATGAAGCACA	CAAACACACCTCTTCTCCACC
MIR31HG	CGCTTCTGTCCTCCTACTCG	ACAAGCAGACCCTTGGAATG
XIST	AGTGCTCTATACGTGGCGGT	ATGCAACCCCAGCAATAGTC
GAS5	CGACTCCTGTGAGGTATGGTG	ATCCTTCCTTGGGGACACAAC
H19	CCTCAAGATGAAAGAAATGGTGCTA	TCAGAACGAGACGGACTTAAAGAA
7SL	GGAGTTCTGGGCTGTAGTGC	ATCAGCACGGGAGTTTTGAC
Gadd7	GGGAAGCTGAGGTTTTTCC	CACACCAGTCTCAACTCCC
HNRNPA1	AAGCAATTTTGGAGGTGGTG	ATAGCCACCTTGGTTTCGTG
FAL1	CCTGGCCAAGAAGCTCATAC	TGAGGACACCGACTACTGAGAA
MIAT	TTTACTTTAACAGACCAGAA	CTCCTTTGTTGAATCCAT
Kcnq1ot1	ATAGTAGTTGGAGACTTCA	ACTGTATATTCAATGTTGGT
TERRA	CAGCGAGATTCTCCCAAGCTAAG	AACCCTAACCACATGAGCAACG
17A	CCACCCTGCAACTGACACAT	GCAAAGGTGCTAATCTTGACTCTTG
Lethe	ACAATGAAGCCAAACTGCCG	AGTTTGTCCAAGGGACCCCA
ANRASSF1	TTACCTCACACTGCTACG	CGATAGAGATCCAACTGT
PAPAS	GCACATCGATACTTAACGTC	GCGTAGCGATGTCGTCCGCAACGGA
HOTAIR	CAGTGGGGAACTCTGACTCG	GTGCCTGGTGCTCTCTTACC
ANRIL	CTGGGACTACAGATGCACCAC	GGAGGGAGCATGTCTGTTTCT
BC200	AGACCTGCCTGGGCAATATAGC	GTTGTTGCTTTGAGGGAAGTTACG
SALNR	GATCTAACTTTTTTTCCTCCT	GAAGTTCTGAGACAACACAAC
TERC	CCGCCTTCCACCGTRCATTC	ACAGAGCCCAACTCTTCGC
MEG3	CCTGCTGCCCATCTACACCTC	CCTCTTCATCCTTTGCCATCCTGG
AS Uchl1	AAACCCATCCTTTCACCATCC	TTCCTATCTTCAGCCACATCAC
BANCR	TTCCTTAGGGTCAGGGGTCT	GATTGGGACCCTTTTCTGGT
PTENpg1	AGTCACCTGTTAAGAAAATGAGAAGACAAA	CTGTCCCTTATCAGATACATGACTTTCAA
HULC	ACCTCCAGAACTGTGATCCAAAATG	TCTTGCTTGATGCTTTGGTCTG
UCA1	TGACATTCTTCTGGACAATGAGTCC	GGCATATTAGCTTTAATGTAGGTGGC
BACE1‐AS	GTAGGCAGGGAAGCTAGTACTGA	AGAGGCTTGCAGTCCAGTTC
MANTIS	GGGTGGGTACACCTGGAATC	ACAGGAGGCTGCTGTCTGAG
TUG1	CATAGTATCATCTTCGGGTTAC	CACAAAATGCATGTAGGTTC
LincRNA‐p21	GGGTGGCTCACTCTTCTGGC	TGGCCTTGCCCGGGCTTGTC

### 4.8 Histological analysis

The fixed hearts were sectioned, and cardiac fibrosis was assessed by staining with Masson's trichrome. Immunohistochemical staining was performed on paraffin‐embedded sections with a primary antibody against CD31 (AF3628, R&D, USA) and BRG1 (SC‐17796, Santa, USA) which was followed by incubation with a biotinylated secondary antibody as described previously (Wu et al., 2017).

### 4.9 Statistics

All the data were expressed as means ± standard error of the mean (SEM) using GraphPad Prism 8. Two‐group comparisons were analyzed by a Student's *t* test. For comparisons of more than two groups, one‐way ANOVA was employed. Statistical significance is indicated by *p < 0.05.

## CONFLICT OF INTEREST

The authors declare no competing interests.

## AUTHOR CONTRIBUTION

QL designed the study. CK, DL, CH, and RL conducted searches, extracted, and analyzed the data. CK and DL wrote the manuscript. All authors contributed to the article and approved the submitted version.

## Data Availability

The data that support the findings of this study are available from the corresponding author upon reasonable request.
